# Around the Hospitals

**Published:** 1893-05-13

**Authors:** 


### Around the Hospitals.

Notice to the Managers of Institutions.—Special space is now reserved for the insertion of notices of hospital meetings and festivals. The Editor requests that all notices may reach The Hospital Office, 428, Strand, at least one week before the date of each meeting.

**The Oldham Infirmary.**—This institution was unfortunately visited by an outbreak of typhoid during the past year, which necessitated a large outlay to remove all cause of infection. This has increased the adverse balance existing on the accounts, and to remove this it is proposed to make special efforts on its behalf. During the year 678 patients were relieved in the hospital. The addition of a special eye department has proved most useful, and already 42 patients have been treated. Twenty-three patients have been sent to convalescent homes, a number adequate to meet requirements in the case of adult patients, but insufficient in the case of children. The Matron, Miss Hunter, who entered the post in October last, has secured the confidence of the House Committee.

**St. George's Hospital.**—A point of interest is brought out in the report of this hospital for 1892 not frequently met with in these yearly records. This is statistics showing the number of patients admitted by letter and through other means. Here we learn that of the 3,517 patients treated in 1892, 2,186 urgent cases were admitted without letters of recommendation, 668 were admitted through governors or subscribers, 54 through the Hospital Sunday Fund, and 17 through the Hospital Saturday Fund. Statements such as these are useful and interesting. They point out in the case of St. George's Hospital the large number of emergency cases such a hospital relieves. The total number of patients admitted during the year show a decrease owing to the fact that the hospital was closed for some time for repairs. These repairs and alterations have been most successfully executed. The new officers' home is now occupied, as is also a nurses' home in Brompton, where probationers are located. Miss Kempton, who has charge of the home, is congratulated on her arrangements. In spite of these improvements there still remains much to be done in arranging the old buildings of the hospital to meet modern requirements, and the surgery and casualty rooms need enlarging. Other points require attention, and a committee has been appointed to consider the matter. The want of funds is, as in the case of many other institutions, the drawback to the work. The extraordinary expenditure of the hospital last year alone amounted to £19,798 out of an income of £31,259. St. George's Hospital possesses two useful accessories to its work in the Samaritan Fund and the Atkinson Morley's Convalescent Hospital. No less than 33 per cent. of the patients treated in the parent institution were removed to the latter to finish their cure. The temporary difficulties under which the home suffered during the year owing to the illness of the Resident Medical Officer were ably met by Miss Sharp, the Matron.

